# Insights gained through real-time monitoring of porcine reproductive and respiratory syndrome virus and description of temporal trends based on laboratory data in Ontario, Canada

**DOI:** 10.3389/fvets.2025.1528422

**Published:** 2025-01-29

**Authors:** Tatiana Petukhova, Maria Spinato, Tanya Rossi, Michele T. Guerin, Cathy A. Bauman, Pauline Nelson-Smikle, Davor Ojkic, Zvonimir Poljak

**Affiliations:** ^1^Department of Population Medicine, Ontario Veterinary College, University of Guelph, Guelph, ON, Canada; ^2^Animal Health Laboratory, Laboratory Services Division, University of Guelph, Guelph, ON, Canada

**Keywords:** porcine reproductive and respiratory syndrome virus, swine, infectious respiratory disease, disease surveillance, real time interactive visualizations, restriction fragment length polymorphism pattern, farm, epidemiological data quality

## Abstract

Porcine reproductive and respiratory syndrome virus (PRRSV) is a prevalent pathogen that impacts the health of swine and is costly to the swine industry. This study utilized PRRSV test results from the University of Guelph's Animal Health Laboratory database to develop interactive, real-time dashboards and to monitor and investigate PRRSV data. The test results from Ontario swine herd samples submitted from January 2014 to July 2023 were processed in R v.4.1.1. The final optimized, aggregated, and anonymized datasets were exported to the Tableau server and were used to design dynamic real-time visualizations with Tableau Desktop v.2021.4. Constructed dashboards were: (1) monthly number of submissions and positive submissions over the last 10 years; (2) number of submissions and positive submissions over the last 3 years, interactively displayed at weekly, monthly, quarterly and yearly intervals; (3) monthly number of PRRSV restriction fragment length polymorphism pattern (RFLP) types at the submission level over the last 5 years; (4) weekly number of tested farms and positive farms over the last 6 Years; (5) monthly number of tested farms and positive farms over the last 6 Years; (6) indicators of the epidemiological data quality in each month; and (7) contextual information. Eighty different PRRSV RFLP patterns were identified with the predominant patterns being 1-8-4, 1-1-1, 1-4-2, and 2-5-2. Most farms contributed one submission per week or per month for PRRSV testing (median: 1 submission per week; IQR: 0; max: 13; median: 1 submission per month; IQR: 1, max: 31). Epidemiological data quality showed considerable improvements over the 9 years of investigation. Apparent changes in trends of submissions were visually observed when time series were stratified by reasons for submission and production class.

## 1 Introduction

Porcine reproductive and respiratory syndrome (PRRS) continues to be a disease of high importance in the commercial swine sector globally. The PRRS virus (PRRSV) can circulate for a long time (1) requiring a strategic approach to minimize clinical signs, and reduce, or eliminate infection (1, 2). The structure and the organization of the commercial swine industry can contribute to the dissemination of infection. For example, when a primary sow herd is infected, the virus will spread to the multiple down-stream sites (i.e., nursery and finisher sites) for some time, through movement of infected animals. Besides animal movement, other transmission pathways can further contribute to spread between farms including contaminated transportation vehicles or shared employees. Although applied in a different context, prevention and disease control rely on improving external and internal biosecurity, immunization, and intensive testing (3, 4). This testing can be done for the purposes of prevention (e.g., maintenance of disease freedom, herd certification, gilt isolation), case confirmation (e.g., initial diagnostic confirmation), and disease control and management (e.g., detection of PRRSV circulation after implementation of disease control measures, gilt acclimation). Characterization of PRRSV strains for tracking disease facilitates decision making on a short- or medium-term basis. Once collected and aggregated over multiple farms, it can serve as an information source to provide insight into longer-term trends. Various approaches have been taken in other jurisdictions to display PRRSV monitoring data in near-real time (5, 6).

The primary objective herein is to describe development of interactive and real-time dashboards to display PRRSV data submitted from Ontario (Canada) swine herds to the Animal Health Laboratory (AHL), University of Guelph. The secondary objective is to describe the PRRSV data, which contributed to the development of interactive dashboards in the period between January 2014 and July 2023.

## 2 Materials and methods

### 2.1 Swine submission form and types of information

Most of the livestock diagnostic testing in Ontario is performed by the AHL. Samples for PRRSV testing were submitted by a veterinarian either at AHL Specimen Reception or sent by Purolator courier (7). Samples were accompanied by a completed swine submission form that may contain information on: the dates of sampling and submission; submitting veterinarian, clinic, and owner information, including the address, contact information, unique premises identifier (ID), and barn group/pen ID; demographic information (e.g., porcine breed, animal age, sex, herd size, commodity, case type, weight, morbidity, mortality, etc.). Commodity groups include sow/gilt, suckling, nursery/weaner, finisher, boars, and other/unspecified. Case types are categorized as diagnostic, monitoring, research, financial and other. Other information may include case history, special instructions, animal. ID, number and type of specimens sent and received, and checkboxes available to indicate types of assays requested. These assays are categorized by laboratory section, i.e., virology, bacteriology, mycoplasmology, parasitology, toxicology, clinical pathology, histopathology, external labs, and other tests requested. Data available on the swine submission form are entered by the staff in AHL Specimen Reception and are combined with the results of tests conducted on the samples that are entered by the laboratory staff in specific laboratory sections of the AHL, resulting in the final test report. Data are organized hierarchically through the unique submission ID and sample ID. For this analysis, swine submissions coming from Ontario and tested for PRRSV using PCR assays were tagged and selected for inclusion in the dashboard and epidemiological analysis.

### 2.2 Data available for analysis

A subset of all available PRRSV data, organized by the submission and sample ID, was obtained from the AHL (8) between January 2014 and July 2023 on swine samples from farms in Ontario, Canada. Every test result was accompanied by submission-level data, which consisted of a unique submission ID, dates when submission was received, sample types, which tests were performed, commodity, purpose of testing (case type), and a description of a diagnostic test. Of relevance for this manuscript and the associated dashboards was a standardized input for commodity and case type. Samples received as part of the individual submissions were tested by PCR to detect PRRSV. At the request of clients, PCR-positive samples within submissions would be further evaluated using sequence analysis of the open reading frame 5 (ORF-5) and to predict the restricted fragment length polymorphism (RFLP) pattern (9). It was done by using the Sanger sequencing.

The AHL received 26,565 unique swine submissions from January 2014 to July 2023 that were tested with PRRSV PCR for diagnostic (83.810%), monitoring (16.096%), research (0.086%), financial (0.004%), or other (not specified) (0.004%) purposes. Submissions tested for financial and research purposes were excluded based on the rationale that they might not represent actual clinical PRRS disease in a herd. The reported test results of tested samples were qualitative (e.g., positive, negative or the typing codes such as 1, 8, 4 based on RFLP characterization). The test results for 6 submissions that were not obtained were excluded. Samples for the remaining 26,535 unique submissions were processed, and the reported test results for each sample were aggregated either at the submission or farm level. In addition, RFLP results for the 1,522 unique positive submissions obtained between January 2019 and July 2023 were retrieved and processed at the submission level. Data processing was executed using R v.4.1.1 (10).

### 2.3 Processing submission-level data

Test results were aggregated at the submission level. The format of qualitative display value was converted into dichotomous: test results reported as “negative” and “not detected” were considered negative; otherwise, they were considered positive. A submission was treated as positive if at least one sample within the submission was declared as positive. The records were cleaned, anonymized, and aggregated (8). Submission dates were converted into weekly intervals starting on Sundays. The number of tested submissions and the number of positive submissions were aggregated by week, case type, and commodity.

### 2.4 Processing farm-level data

Depending on the needs of a farm, more than one submission could be submitted within a week or month for testing for PRRSV, which can inflate the extent of testing in a population if a repeat submission is considered equivalent to the initial submission from an individual farm. Thus, for those submissions that provided accurate premises ID and were submitted from Ontario farms, the number of submissions per unique premises per time interval (i.e., week, and month) was summarized using descriptive statistics. Thereafter, the selected submissions were processed (8) and aggregated by the premises ID within a given time interval. A farm (i.e., premises) was considered tested in each time interval if one or more of the submissions from the farm were tested in that time interval (i.e., a week or a month). Similarly, a farm was treated as positive if at least one submission within the farm was declared positive in that time interval. The number of farms included in the analysis depended on the availability of accurate information for the unique farm identifiers. Therefore, a separate dashboard that allowed for monitoring of the quality of epidemiological data was developed, and the process is explained below.

### 2.5 PRRSV ORF5 typing

PRRSV typing was done by comparing full ORF5 nucleotide sequences and by RFLP analysis (11, 12). RFLP patterns are a code consisting of three numbers (e.g., RFLP 1-8-4) based on the presence or absence of sites for three restriction enzymes (*Mlu*I, *Hinc*II, and *Sac*II). The resulting patterns were made available as a part of the data stream for the dashboards. Each included submission contained at least one sample.

In total, there were 1,522 observations related to RFLP codes. Qualitative results related to RFLP typing (e.g., could not type, negative, not available, unable to type, undetermined, too weak to type) were excluded, resulting in 1427 unique submissions. Submissions that were characterized by several RFLP patterns were classified as “mixed”. Submissions classified into RFLP patterns were processed and the number of submissions was aggregated by week.

### 2.6 Epidemiological indicators of data quality

The availability of demographic data (e.g., age of animals, sex) and premises ID for tested submissions were considered as indicators to monitor the epidemiological quality of the PRRSV data. Three dichotomous variables were created to indicate epidemiological data quality: whether a tested submission provided commodity (demographic information); whether premises ID was available; and whether the provided premises ID was valid. A tested submission had a valid premises ID if the string pattern in the ID was clearly indicative of the Ontario location, i.e., ON followed by several numbers. Submission dates were converted into monthly intervals. The number of tested submissions was aggregated by submission date and by the three created epidemiological dichotomous variables.

### 2.7 Dashboards

The cleaned, anonymized and aggregated test results were stored in the AHL laboratory information management system (LIMS). The data were exported to the Tableau server and made available to Tableau Desktop v.2021.4 (13), where the data fields were connected to the data source with a live connection to directly update any changed fields. The variables of the datasets were used to design real-time visualizations. The visualizations included interactive and dynamic dashboard features, displaying summarized aggregated test results in a concise and informative way. The final versions of constructed dashboards were uploaded to the Ontario Interactive Animal Pathogen Dash- boards (IAPD) website and can be accessed at https://iapd.lsd.uoguelph.ca/. Access to the site is available for everyone who has Internet access. However, the dashboard visualizations are securely stored and can be accessed only with an approved login account. The constructed dashboards are updated daily overnight; however, the data are provided with a 24-h delay to allow data to be transferred among different tables in the database and data processing.

The dashboards can be accessed with an approved login account and are available for licensed veterinarians, government organizations involved in agriculture, public health, researchers, and industry/surveillance partners.

## 3 Results

### 3.1 Overall display of PRRS dashboard

The PRRSV dashboard story has been organized into seven distinct pages, each displaying contextually similar information. This includes: (1) monthly number of submissions, positive submissions, and percent positivity over the last 10 years; (2) number of submissions, positive submissions, and percent positivity over the last 3 years, interactively displayed at weekly, monthly, quarterly and yearly intervals; (3) number of PRRSV RFLP types per month at the submission level over the last 5 years; (4) weekly number of tested farms and positive farms over the last 6 years; (5) monthly number of tested farms and positive farms over the last 6 years; (6) indicators of the epidemiological quality on a monthly level; and (7) contextual information.

### 3.2 Time series of submissions and positive submissions

**Figure 1:**
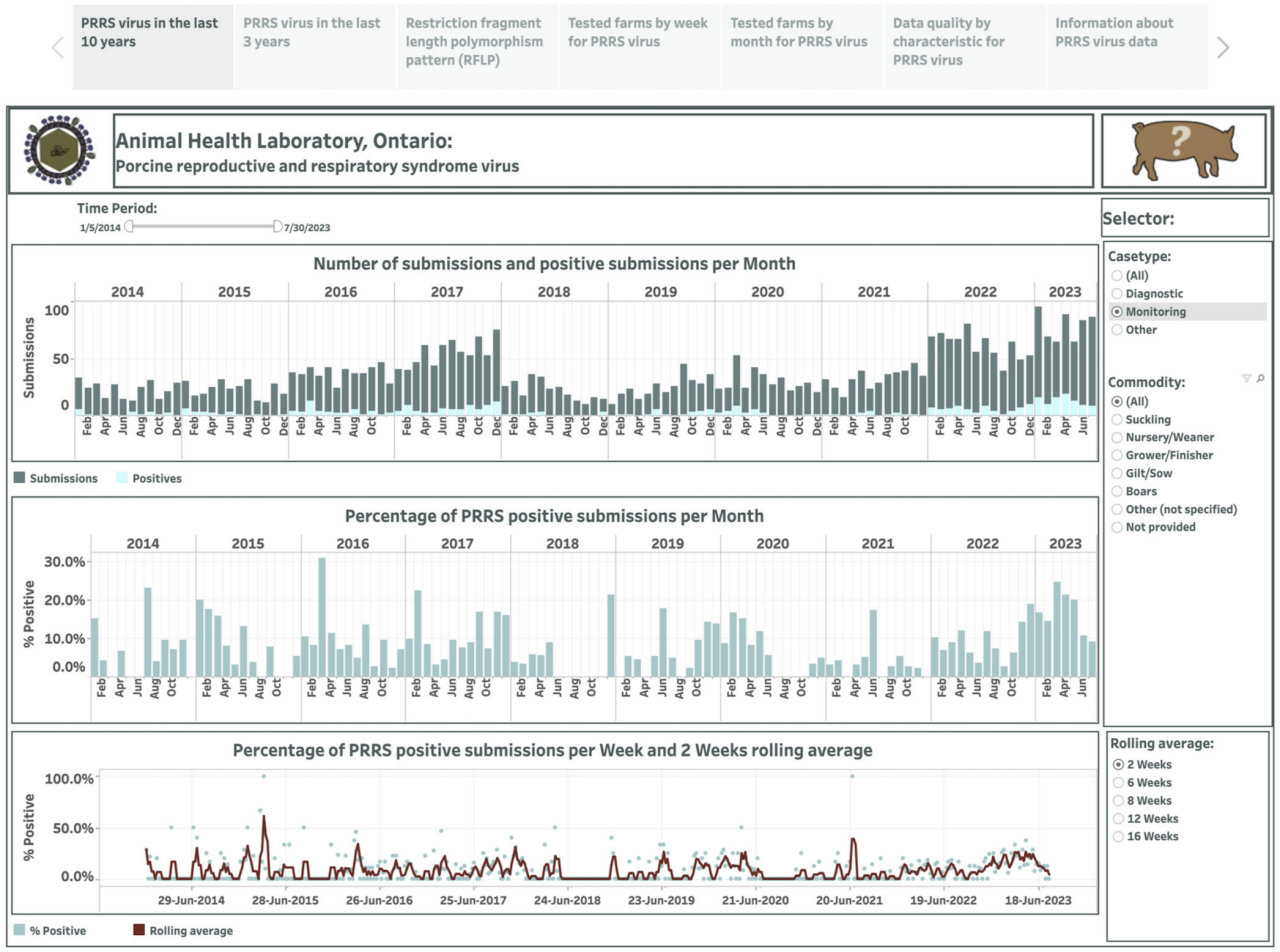
Dashboard for monthly number of submissions for porcine reproductive and respiratory syndrome virus (PRRSV) and positive submissions based on real-time RT-PCR testing over the past 10 years. Figures were built using the number of submissions and positive submissions, where counts were obtained from swine samples submitted to the Animal Health Laboratory in Ontario from January 2014 to July 2023. Charts display aggregated test results obtained from submissions tested for monitoring purpose.

Overall, 499 weekly observations were used to display the occurrence of PRRSV over the past 10 years in the dashboard (Figure 1). The dashboard presented in the first story point summarizes test results across case type and commodity categories monthly from January 2014 to July 2023. The selection of a category is displayed on the right side in Figure 1. The number of submissions and positive submissions are shown with a stacked vertical bar chart where the top bars in dark color represent submission counts, and the bottom bars in light color represent positive submission counts (the top graph in Figure 1). The calculated percentage of positive submissions over the total number of tested submissions in each month is displayed in a vertical bar graph (the middle graph in Figure 1). The computed percentage of positive submissions and estimated moving averages are shown in a dual chart with a scatter plot representing the percentage of positive submissions, and a line representing the rolling average values (the bottom graph in Figure 1). The selection of smoothing degree based on the previous 2, 6, 8, 12, or 16 weeks is displayed in the lower-right corner in Figure 1.

Total monthly submissions ranged from 109 (in April 2014) to 376 (in January 2022), increasing more than two-fold from an average of 133 per month in 2014 to an average of 291 per month in 2023. As the number of tested submissions for PRRSV increased over the years, there was also an increase in the number of positive cases, ranging from 9 (in March 2014) to 85 (in April 2023) and increasing more than 3-fold from an average of 17 per month in 2014 to 59 per month in 2023. The percentage of PRRSV-positive submissions per month ranged from 5.5% to 29.0% from 2014 to 2016, respectively, and then fluctuated around 10% from 2017 onwards. The percent-positive time series is smoothed with a lag of 2 weeks, exhibiting some variations around the average of 13.9%, where the majority of percent positives were higher than the mean from January 2014 to June 5, 2014, and from December 2019 to July 2023. Differences in percent positivity existed when the monthly time series were stratified by the reasons for submission, with 15.2% and 9.0% submissions positive for diagnostic and monitoring purposes, respectively. In addition, when evaluated monthly, submission positivity ranged between 5.6% and 28.8% for diagnostic submissions, and between 0% and 31% for monitoring submissions. Time series based on monitoring submissions had occasional periods when no positive submissions were detected, and this lasted from 1 month to 6 months starting in June in 2018 (Figure 1). In addition, a pattern in the number of monitoring submissions submitted per month was observed, where three distinct periods were visible (Figure 1) with abrupt changes in the number of submissions that coincided with the time that the calendar year ends and starts. The period until 2017 was characterized by a progressively higher number of submissions over time (mean = 38.7, sd = 15.9), followed by a generally lower number of submissions between January 2017 and December 2021 (mean = 27.3 sd = 9.7), and a subsequent increase in the number of monitoring submissions starting in January 2022 (mean = 65, sd = 14.7) (Figure 1). Similar patterns were noted in monitoring submissions in both nursery (Supplementary Figure 1) and grower/finisher pigs (Supplementary Figure 2). An interesting pattern was also observed among submissions for suckling pigs: while the number of all submissions increased over time and particularly since the start of 2020 (Supplementary Figure 3), the number of monitoring submissions in this production class was low until 2022 (Supplementary Figure 4). For example, in 2021, there were a total of 9 monitoring submissions in suckling pigs, whereas the monthly average between January 2022 and the end of the study period was 4.7 (sd = 3.1) (Supplementary Figure 4). Submission number and positivity for boars also showed an interesting pattern, with an upward trend in the number of submissions, but with a small percent positivity (Supplementary Figure 5).

**Figure 2:**
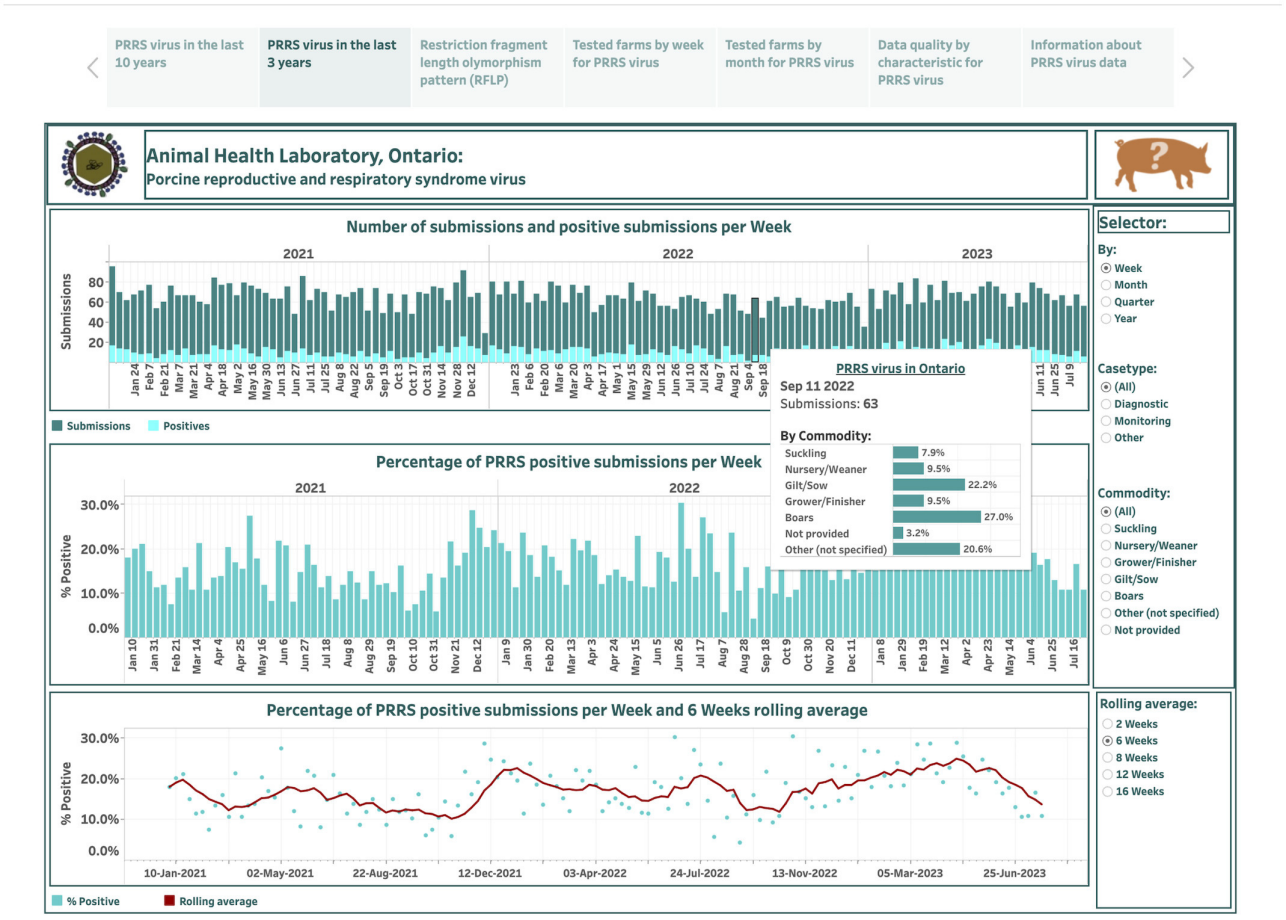
Dashboard for the number of submissions for porcine reproductive and respiratory syndrome virus (PRRSV) and positive submissions based on real-time RT-PCR testing over the past 3 years. Figures were built using the number of submissions and positive submissions, where counts were obtained from swine samples submitted to the Animal Health Laboratory in Ontario from January 2021 to July 2023.

The dashboard describing PRRSV submissions over the last 3 years is designed based on 134 observations and is displayed in the second story point (Figure 2). The displayed features of this dashboard are like the dashboard that summarizes PRRSV data over the past 10 years, except for the aggregation feature. Herein, the test results are aggregated not only across case type and commodity levels, but also at weekly, monthly, quarterly, and yearly time intervals that extend only from January 2021 to July 2023.

Total weekly submissions ranged from 29 on December 26, 2021, to 95 on January 3, 2021, and decreased from an average of 67 per week in 2021 to 66 per week in 2023. Total weekly positive submissions ranged from 2 on September 4, 2022, to 26 on December 5, 2021, and increased from an average of 10 per week in 2021 to 14 per week in 2023. The percentage of PRRSV positive submissions per week ranged from 4.2% on September 4, 2022, to 30.4% on October 30, 2022. The degree of smoothing short-term variation in the percent-positive time series is 6 weeks (Figure 2). From visual inspection, the percent-positive time series appear to behave in a cyclical manner.

### 3.3 Molecular typing of PRRSV

**Figure 3:**
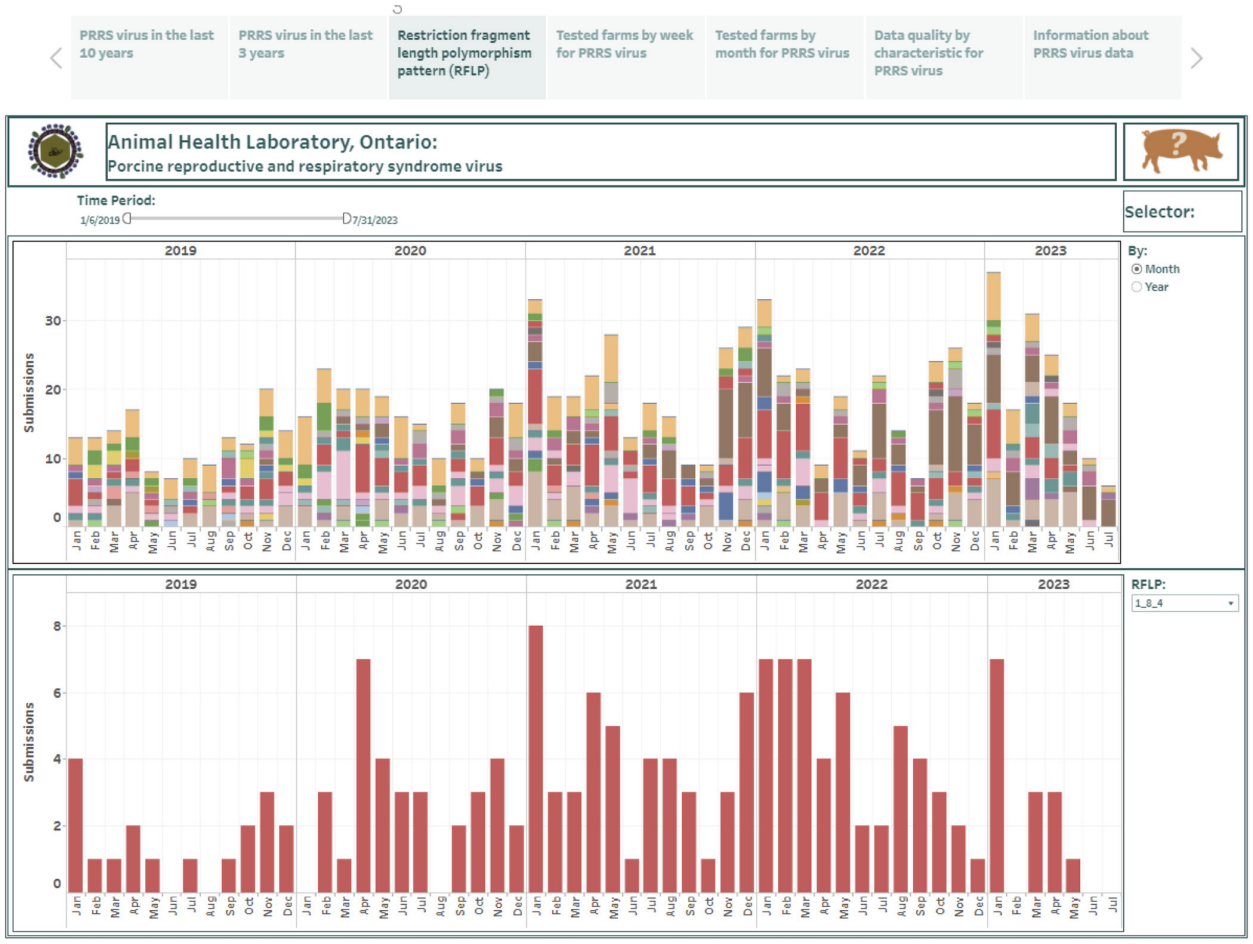
Dashboard for restriction fragment length polymorphism pattern (RFLP) over the past 5 years, summarizing at the submission level. Figures were built using the number of positive submissions, where counts were obtained from swine samples submitted to the Animal Health Laboratory in Ontario and tested for porcine reproductive and respiratory syndrome virus (PRRSV) from January 2019 to July 2023. The counts were classified into RFLPs, predicted using the open reading frame 5 (ORF5) sequence.

The RFLP data consisted of test results from 753 weekly records over a 5-year period (January 2019–July 2023) and was used to construct the restriction fragment length polymorphism (RFLP) pattern dashboard. The RFLP dashboard is presented in the third story point and summarizes the predicted RFLP patterns in PRRSV positive submissions at either monthly or yearly time aggregations (Figure 3). The RFLP pattern counts are presented in a stacked vertical bar graph where each color represents a different detected PRRSV strain (the top graph in Figure 3). A trend for each predicted PRRSV strain can be visualized individually in a vertical bar graph (the bottom graph in Figure 3). The presented test results can be displayed in greater detail by selecting a shorter time interval in the time filter on the top band in Figure 3.

Over the study period considered in this paper, a total of 80 distinct RFLPs were identified. Over the last 5 years, the RFLPs 1-8-4 (16.6%), 1-1-1 (16.2%), 1-4-2 (14.2%), and 2-5-2 (12.5%) constituted 59.5% of all RFLPs identified. The dashboard allowed for visualization of shifts in the occurrence over time. For example, RFLP 1-4-2 was identified in one submission in 2019 while it was detected in 33 submissions in 2023; RFLP 1-1-1 was detected in 34 submissions in 2019, but this RFLP has been identified in 33 submissions in the first 6 months of 2023. Interestingly, 7 submissions were detected with mixed RFLP types over the study period.

### 3.4 Time series of submissions and positive premises

**Figure 4:**
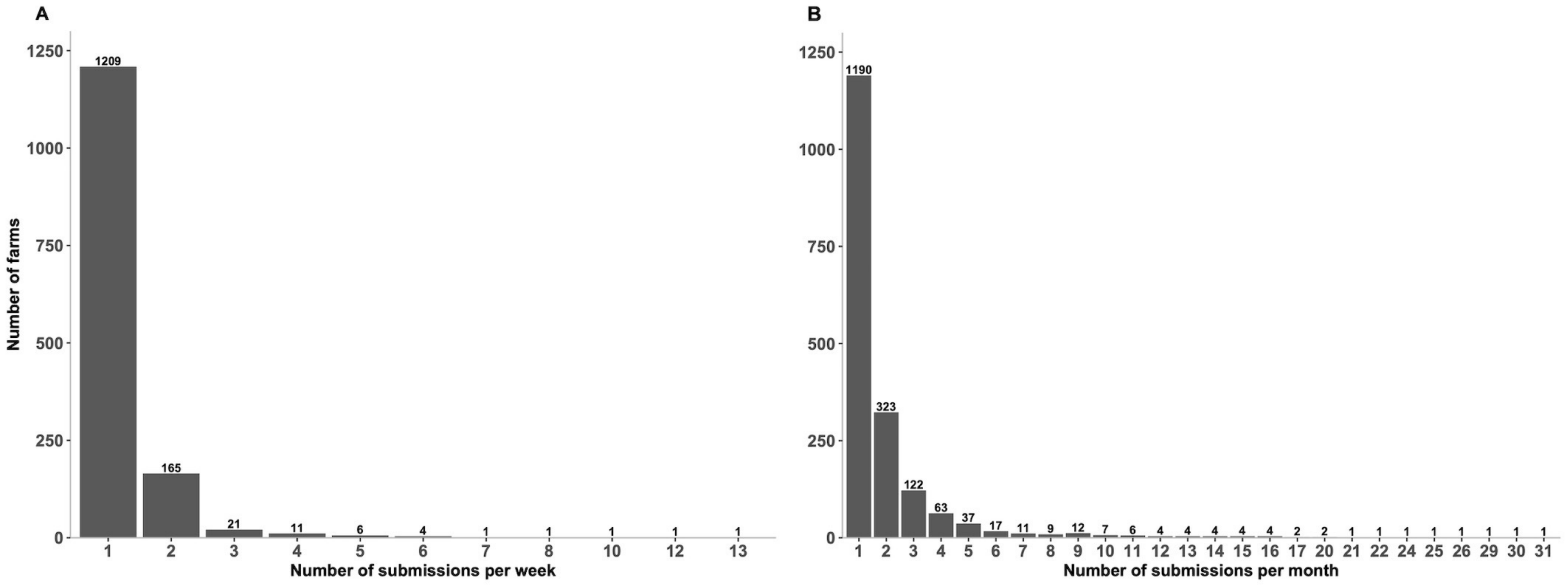
Number of submissions submitted by farms for porcine reproductive and respiratory syndrome virus (PRRSV) based on real-time RT-PCR testing over the past 6 years. **(A)** Number of submissions submitted by farms per week. **(B)** Number of submissions submitted by farms per month. Figures were built using the number of submissions, where counts were obtained from swine samples submitted to the Animal Health Laboratory in Ontario from January 2018 to July 2023. Summary of submitted submissions per week: median = 1; IQR = 0; max = 13. Summary of submitted submissions per month: median = 1; IQR = 1, max = 31.

Overall, 13,299 unique submissions from 1221 premises were submitted over the period January 2018 to July 2023. The descriptive statistical analysis reveals that 1,209 farms (99%) submitted one submission per week (median = 1; IQR = 0; max = 13) (Figure 4A), and 1,190 farms (97.5%) submitted one submission per month (median = 1; IQR = 1, max = 31) (Figure 4B). Furthermore, 165 farms (13.5%) submitted two submissions per week (Figure 4A) and 323 farms (26.5%) submitted two submissions per month (Figure 4B).

**Figure 5:**
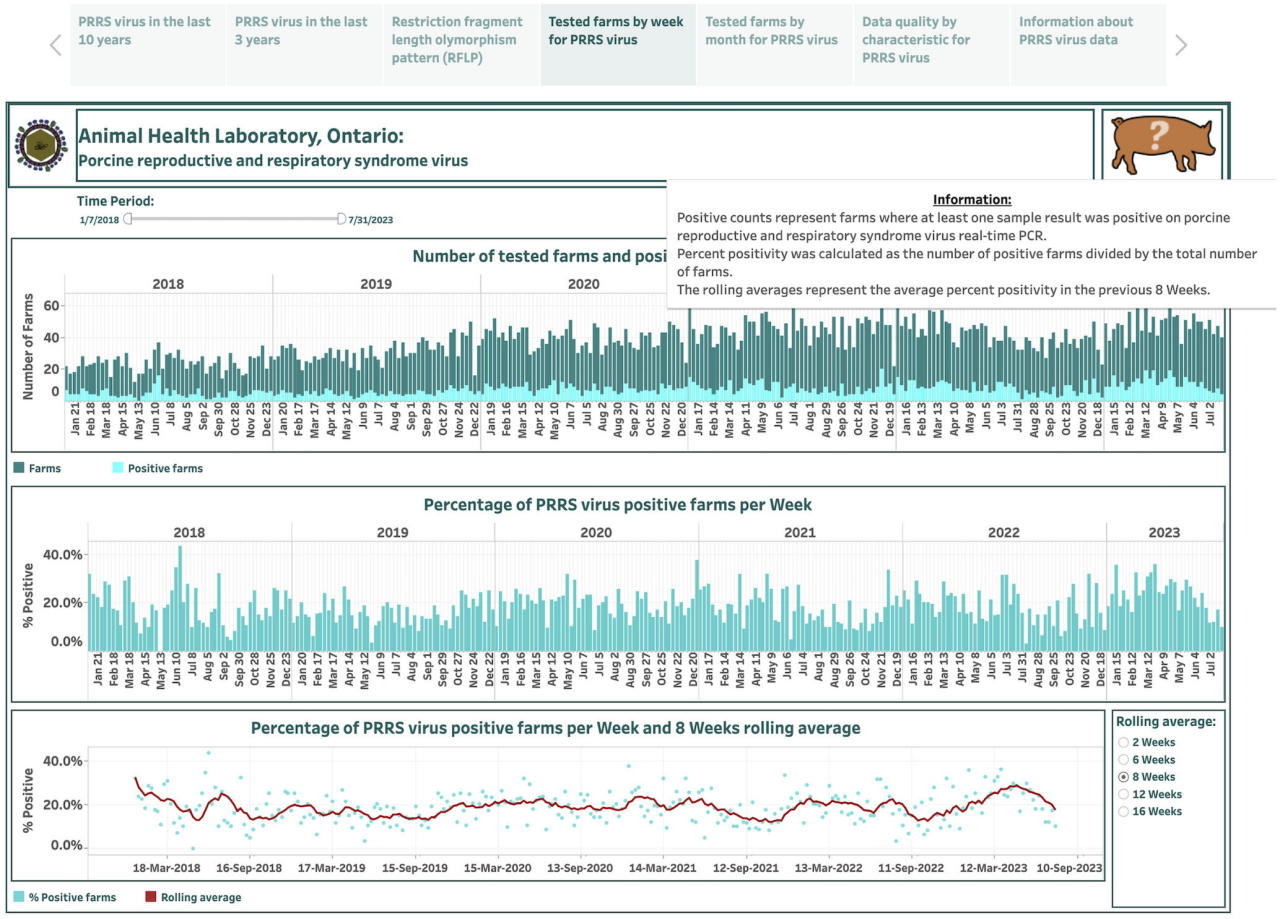
Dashboard for weekly number of farms for porcine reproductive and respiratory syndrome virus (PRRSV) and positive farms based on real-time RT-PCR testing over the past 6 years. Figures were built using available premises IDs, where counts were obtained from swine samples submitted to the Animal Health Laboratory in Ontario from January 2018 to July 2023.

**Figure 6:**
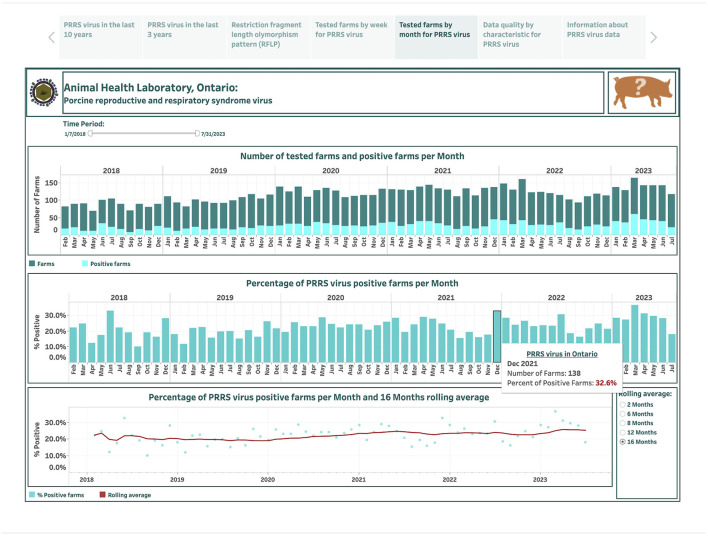
Dashboard for monthly number of farms for porcine reproductive and respiratory syndrome virus (PRRSV) and positive farms based on real-time RT-PCR testing over the past 6 years. Figures were built using available premises IDs, where counts were obtained from swine samples submitted to the Animal Health Laboratory in Ontario from January 2018 to July 2023.

The dashboards describing PRRSV farm-level submissions over the last 6 years are designed based on 290 weekly and 67 monthly records and are presented in the fourth and fifth story points, respectively (Figures 5, 6). An animal icon on the top band provides brief information about counts and how calculations were made (the upper-right corner in Figure 5). The number of tested farms and positive farms are presented by a stacked vertical bar chart (the top graph in Figures 5, 6). The top bars in dark color represent farm counts, while the bottom bars in light color represent positive farm counts (Figures 5, 6). The calculated percentage of positive farms over the total number of tested farms in each week or month is shown in vertical bar graphs (the middle graph in Figures 5, 6). The computed percentage of positive farms and estimated moving averages are displayed in dual charts with a scatter plot and a line, respectively (the bottom graph in Figures 5, 6). The selection of smoothing degree based on the previous 2, 6, 8, 12, or 16 weeks/months is given in the lower-right corner in Figures 5, 6. Hovering the cursor over any bar displays additional detailed information about counts (Figure 6).

The total number of farms tested each week ranged from 12 on May 6, 2018, to 64 on April 23, 2023, increasing from an average of 24 per week in 2018 to 48 per week in 2023 (Figure 5). The total number of positive farms ranged from 0 on May 13, 2018, to 20 on December 5, 2021, increasing from an average of 5 per week in 2018 to 11 per week in 2023. The percentage of PRRSV positive farms per week ranged from 0% on May 13, 2018, to 43.2% on June 17, 2018. The degree of smoothing short-term variation in the percent-positive time series is 6 weeks. From visual inspection, the average does not fluctuate notably as it moves with the data.

The total number of farms tested each month ranged from 69 in May 2018 to 164 in March 2023, increasing from an average of 86 per month in 2018 to 139 per month in 2023 (Figure 6). The total number of positive farms ranged from 7 in September 2018 to 60 in March 2023, increasing from an average of 18 per month in 2014 to 40 per month in 2023. The percentage of PRRSV positive farms per month ranges from 10% in September 2018 to 36.6% in March 2023. A lag of 16 months is selected to smooth the percent-positive time series. A visual inspection suggests a slowly increasing trend in percent positivity over 6-year time period.

### 3.5 Indicators of epidemiological data quality

**Figure 7:**
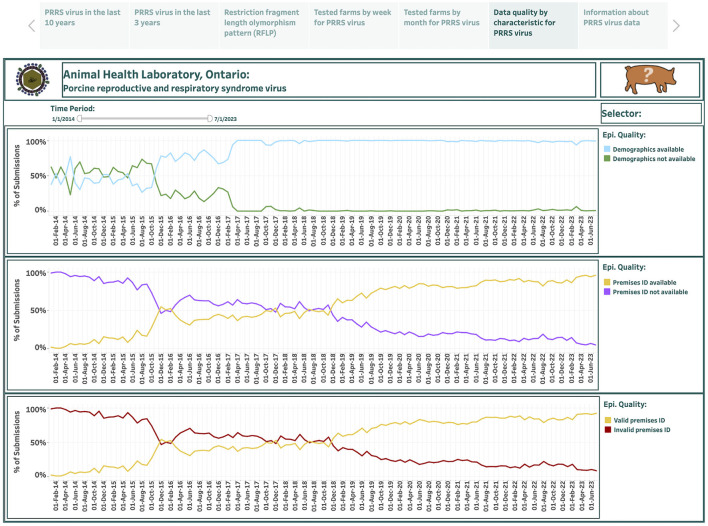
Dashboard for data quality by variable for porcine reproductive and respiratory syndrome virus (PRRSV), test submissions over the past 10 years. Figures were built using the number of submissions, where counts were obtained from swine samples submitted to the Animal Health Laboratory in Ontario from January 2014 to July 2023 and assessed for the epidemiological data quality.

Overall, 115 monthly records were used to describe the data quality by characteristic for PRRSV test submissions in the last dashboard spanning 10 years. This dashboard is presented in the sixth story point and summarizes the percentage of submissions for three epidemiological data quality variables from January 2014 to July 2023: the availability of demographic information (commodity level); the availability of premises ID; and the availability of valid premises ID (Figure 7). The calculated percentage of submissions that provided demographic information over the total number of tested submissions in each month is shown in a lightly colored line, while the calculated percentage of submissions that did not specify demographic information over the total number of tested submissions in each month is shown in a darker line (the top graph in Figure 7). The calculated percentage of submissions that provided premises ID over the total number of tested submissions in each month is shown in a lightly colored line, while the calculated percentage of submissions that did not identify premises ID over the total number of tested submissions in each month is shown in a darker line (the middle graph in Figure 7). The calculated percentage of submissions that provided valid premises ID over the total number of tested submissions in each month is shown in a lighted colored line, while the calculated percentage of submissions that either did not provide premises ID or provided invalid premises ID over the total number of tested submissions in each month is shown in a darker line (the bottom graph in Figure 7).

From January 2014 to July 2023, the percentage of submissions that had available demographic information increased from 37.8% to 99.2%; the percentage of submissions that provided premises IDs increased from 1.3% to 95.7%; and the percentage of submissions that provided valid premises IDs increased from 1.3% to 92.2%.

### 3.6 Brief information regarding PRRSV data

The last story point provides brief information regarding PRRSV data. The information page describes the effects of PRRSV on pig health, clarifies how submissions were processed, and explains how computations were performed.

## 4 Discussion

This paper describes the design of a web-based tool that displays PRRSV aggregated test results at the submission level, findings from the RFLP typing, and demographic data to display the frequency of PRRSV in Ontario. There are several swine surveillance reporting systems available in Canada (14–17), and this web-based visualization tool is an additional information source providing comprehensive real time PRRSV test results. Previously published reporting systems have demonstrated the value of implementing web-based tools (5, 17–20). Considering the usefulness of these tools and the users' need for real-time visualizations of trends for major pathogens, we used R for PRRSV data preparation and implemented Tableau for presenting PRRSV data in different graphical forms and executing real-time data updates. By integrating these software features, we processed, analyzed, and presented the massive and complex animal pathogen test results data derived from the AHL in real-time visualizations.

Our constructed visualizations were embedded in the password-protected visualization platform and uploaded online, accessible through an approved login account. We employed features that highlight key points, aggregate data across purpose of testing and commodity level at different time intervals, allow users to interact with charts, and provide explicit explanations to help users comprehend graphics and gather knowledge about the actual PRRSV situation on Ontario swine farms. The visualizations are presented as a story based on logical reasoning, where each page is a dashboard that summarizes the PRRSV data from a general perspective to a more detailed analysis portraying different aspects. In our approach, we dedicated a separate dashboard to monitor the epidemiological quality of the data. In our view, statistics that indicate epidemiological or other aspects of data quality are necessary for knowledgeable end users to make proper conclusions about the visualizations. For example, in the case that a substantial proportion of submissions do not provide information about premises identification, calculation of PRRSV-positive farms would be based only on a subset of data where such information was provided. A calculation of percent positive farms from such data may not be biased if this subset is a true random sample of the entire source population of farms tested. Nonetheless, if the reason for not submitting premises IDs is linked with the outcome, biased results in the disease frequency at the farm level would likely occur. Similarly, if the reason for not providing demographic information is associated with a specific demographic strata and test positivity, the visualization based on the demographic strata would likely yield a biased interpretation of positivity in different strata. With our approach to data quality, the end user can make their own assessment about this aspect of data quality. Additionally, providing such statistics serves the purpose of informing the entire source population of veterinary practitioners whether standard operating procedures and good practices are being followed by most participants. At the time of writing, the indicators of quality for PRRSV data were all above 98%, which provides confidence about the completeness of the data. It also serves as an example of what can be done with such information, while maintaining privacy of participating farms, a common concern in some industries that are hesitant to provide such information. Indicators of data quality should be interpretable by the intended users, and in our case, individuals with knowledge of their source populations and industries. Previous studies that summarized experiences of developers who developed COVID-19 dashboards reported issues with misinterpretation of visualizations for statistics that were accessible to general public (21). To minimize this risk, a commonly reported strategy in the latter study was to contextualize information presented on dashboards. For dashboards developed and reported herein, we used several approaches to minimize misinterpretation. First, we made this potentially sensitive information accessible only to experts. Secondly, we contextualized information to a degree that our resources allowed. Thirdly, we provide data quality indicators where feasible. We recommend that different indicators of data quality should be presented as an integral part of the dashboards and should be monitored over the displayed time frame (14–17).

Through exploratory analysis of available dashboards and stratification features built into dashboards several large-scale patterns could be observed, suggesting some trends in the intensity of surveillance, and possibly some alterations in surveillance and testing strategies. For example, there was an increase in the number of PRRSV test submissions over time. The methodology in this study does not allow us to speculate about all the reasons for such trends; however, this trend suggests changing attitudes toward the importance of PRRSV testing in swine health management and decision making. Decisions made at the level of individual farms were illustrated by the number of submissions per farm per week and month, which in extreme cases were indicative of daily testing in some periods. Another interesting pattern was obvious changes in the numbers of monitoring submissions that coincided with the start and the end of the calendar year. Typically, submissions submitted for diagnostic reasons should be indicative of confirmatory testing following a clinical issue, whereas monitoring should be indicative of testing for the purpose of early identification of a subclinical infection or for certification purposes. Research into understanding how end users define a monitoring vs. a diagnostic submission, and whether they adhere to their definition when submitting samples for pathogen testing, is warranted before relying too much on its interpretation. Classification into monitoring and diagnostic submissions as the two basic categories is sensible, but it would be prudent to understand practices that contribute to defining these submission types, and motivations of people who submit samples to indicate certain submission type. This could be particularly useful for pathogens where specific guidelines for diagnostic testing and ongoing monitoring do not exist or there is no universal acceptance of using the existing recommendations. Standardization of these basic terms and the widespread acceptance and implementation of guidelines for monitoring and disease certification would also be beneficial if a more formal statistical comparison of trends based on monitoring and on diagnostic submissions is attempted. Abrupt changes observed descriptively in this study, especially in some demographic strata, that coincided with the start and end of the year are suggestive of changes in the processes and best practices in the industry, which are not necessarily widely known. Another interesting trend was the high number of monitoring submissions in suckling piglets. This submission frequency may reflect the widespread use of processing fluids for monitoring (22, 23). There was a high volume of boar testing with a low percent positivity as most breeding boars are screened for PRRSV in their semen. The best practices in the swine industry since 2011 are to routinely monitor boar studs to ensure they are negative for PRRSV (24, 25). Quick examination of periodicity evidence in the percentage of PRRSV positive submissions did not seem to suggest anything that could have biological rationale.

Overall, these examples suggest that testing for PRRSV is complex, and there are many factors that may influence submission patterns. The results from the RFLP analysis were integrated into the development of the dashboards. Eighty different RFLP patterns were identified over the 5-year period. The predominant patterns were 1-8-4, 1-1-1, 1-4-2, and 2-5-2, which represented about 12%−16% of strains. The latter pattern (i.e., 2-5-2) can be classified as a vaccine-like pattern, one of the four vaccine-like patterns for four vaccine products currently licensed for PRRSV in Canada. The same method was used in a study conducted in Ontario from 1998 to 2000, and it was reported that 34 different patterns were identified from PRRSV strains (26). At that time 1-8-4 PRRSV was not detected. A study conducted in Ontario in 2021 reported that 1-8-4, 1-1-1, 1-4-2, and 2-5-2 were among the top 11 RFLP designations (27). In Québec it was reported that a total of 29 RFLP patterns (1998–2002) were obtained from sequencing of PRRSV strains, also with predominant 1-8-4 and 2-5-2 patterns among others (28). Although it has been reported in the literature that RFLP patterns are inaccurate representations of molecular typing because of some methodological caveats (11, 29), this technique is still widely used for differentiation of strains. While ORF5-based genetic lineages and sublineages classification systems for PRRSV have been explored (27, 30, 31) and have become widely used nowadays (32, 33), the output of such classification was not available in the database used to generate the dashboards and could not be displayed. Nonetheless, when available, such designations could be used alone in this dashboard, or in combination with phylogenetic trees to display genotyping PRRSV information.

This is a descriptive study that aimed to describe development of interactive and real-time dashboards to display PRRSV data submitted from Ontario swine herds to the Animal Health Laboratory, to describe the PRRSV data that contributed to dashboards, and to illustrate how some stratifications and features in the dashboards could be used to explore the data. Through such explorations some descriptive trends could be observed, and recommendations could be made about further strengthening of PRRSV surveillance. Monitoring data quality, possibility of providing farm-level in addition to submission-level disease frequency over time, standardization of how diagnostic vs. monitoring submissions are labeled, and reporting of genotyping information and their further refinement are useful not only for PRRSV surveillance but likely for many other production-limiting diseases in different livestock and poultry populations. Unlike our previous attempts (34), we did not attempt to evaluate the observed trends using statistical inference approaches, but this study and the interactive dashboards that were developed as a part of it could serve as a basis to explore ongoing large-scale trends and initiate further quantitative, qualitative, and forecasting studies.

The limitations of this study are inherent to the type of data we used. It is a passive surveillance system that relies on submissions from the field and as such is prone to different types of biases and changes in the industry, surveillance, and testing approaches. Addressing some of the observations and recommendations made in this study could help alleviate these biases; nonetheless, the best use of such laboratory data is when the data are integrated with other types of information and contextualized by experts. One such example is the Ontario Animal Health Network, which also can utilize information from the dashboards present herein (35).

The use of RFLP to track genotyping information has severe limitations. Future work is needed to provide ongoing analysis and classification into lineages/sublineages based on reference sequences used in other studies (33). This work would include a thorough analysis and exploration of classification based on such reference sequences in the target population of PRRSV ORF-5 sequences in Ontario and Canada.

## Data Availability

The datasets presented in this article are not available for reasons of confidentiality. Reasonable requests to access the aggregated data should be directed through the IAPD website (https://iapd.lsd.uoguelph.ca/).

## Supplementary material

Supplementary Figure 1 Dashboard for monthly number of submissions for porcine reproductive and respiratory syndrome virus (PRRSV) and positive submissions based on real-time RT-PCR testing for nursery/weaner pigs over the past 10 years. Figures were built using the number of submissions and positive submissions, where counts were obtained from swine samples submitted to the Animal Health Laboratory in Ontario from January 2014 to July 2023. Charts display aggregated test results obtained from submissions tested for monitoring purpose.

Supplementary Figure 2 Dashboard for monthly number of submissions for porcine reproductive and respiratory syndrome virus (PRRSV) and positive submissions based on real-time RT-PCR testing for grower/finisher over the past 10 years. Figures were built using the number of submissions and positive submissions, where counts were obtained from swine samples submitted to the Animal Health Laboratory in Ontario from January 2014 to July 2023. Charts display aggregated test results obtained from submissions tested for monitoring purpose.

Supplementary Figure 3 Dashboard for monthly number of submissions for porcine reproductive and respiratory syndrome virus (PRRSV) and positive submissions based on real-time RT-PCR testing for suckling pigs over the past 10 years. Figures were built using the number of submissions and positive submissions, where counts were obtained from swine samples submitted to the Animal Health Laboratory in Ontario from January 2014 to July 2023 for all case types.

Supplementary Figure 4 Dashboard for monthly number of submissions for porcine reproductive and respiratory syndrome virus (PRRSV) and positive submissions based on real-time RT-PCR testing for suckling pigs over the past 10 years. Figures were built using the number of submissions and positive submissions, where counts were obtained from swine samples submitted to the Animal Health Laboratory in Ontario from January 2014 to July 2023. Charts display aggregated test results obtained from submissions tested for monitoring purpose.

Supplementary Figure 5 Dashboard for monthly number of submissions for porcine reproductive and respiratory syndrome virus (PRRSV) and positive submissions based on real-time RT-PCR testing for boars over the past 10 years. Figures were built using the number of submissions and positive submissions, where counts were obtained from swine samples submitted to the Animal Health Laboratory in Ontario from January 2014 to July 2023 for all case types.
