# Supplementary figures and images for: Insights gained through real-time monitoring of porcine reproductive and respiratory syndrome virus and description of temporal trends based on laboratory data in Ontario, Canada
Source: Front Vet Sci. 2025 Jan 29;12:1528422. doi: 10.3389/fvets.2025.1528422 (PMC11813902; doi:10.3389/fvets.2025.1528422)

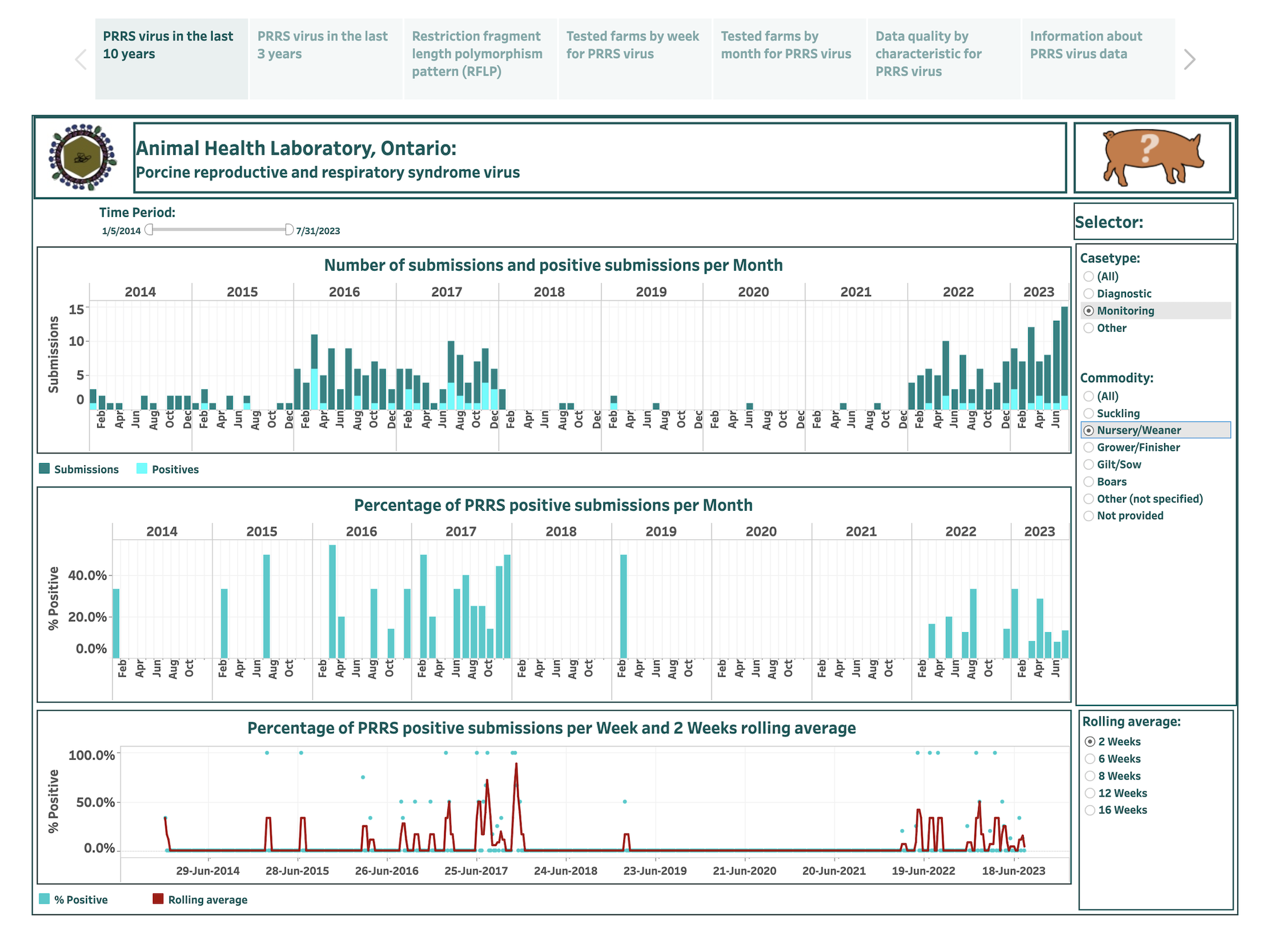

Supplement: Supplementary Figure 1 — Dashboard for monthly number of submissions for porcine reproductive and respiratory syndrome virus (PRRSV) and positive submissions based on real-time RT-PCR testing for nursery/weaner pigs over the past 10 years. Figures were built using the number of submissions and positive submissions, where counts were obtained from swine samples submitted to the Animal Health Laboratory in Ontario from January 2014 to July 2023. Charts display aggregated test results obtained from submissions tested for monitoring purpose. [file Image_1.tiff]

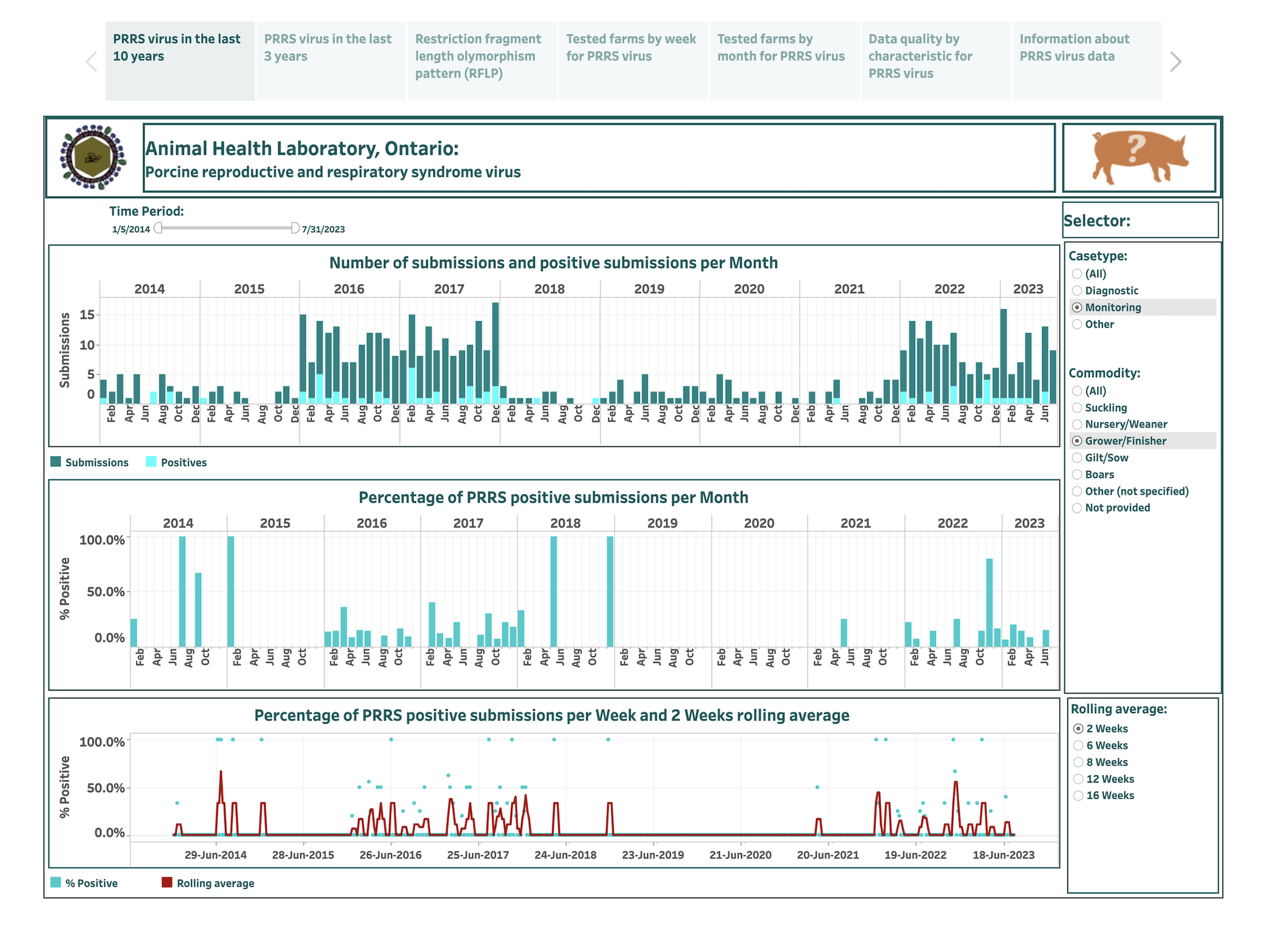

Supplement: Supplementary Figure 2 — Dashboard for monthly number of submissions for porcine reproductive and respiratory syndrome virus (PRRSV) and positive submissions based on real-time RT-PCR testing for grower/finisher over the past 10 years. Figures were built using the number of submissions and positive submissions, where counts were obtained from swine samples submitted to the Animal Health Laboratory in Ontario from January 2014 to July 2023. Charts display aggregated test results obtained from submissions tested for monitoring purpose. [file Image_2.tiff]

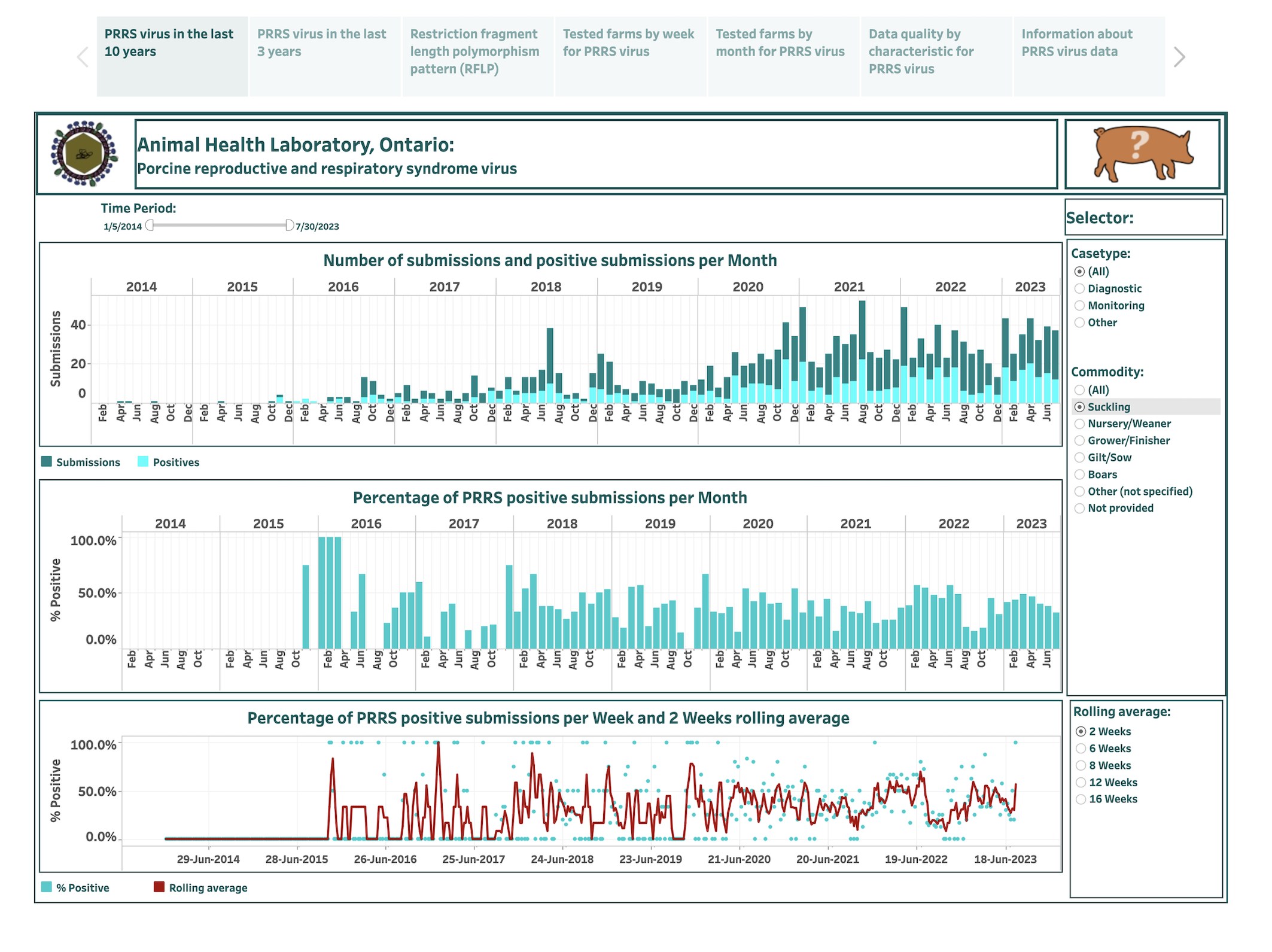

Supplement: Supplementary Figure 3 — Dashboard for monthly number of submissions for porcine reproductive and respiratory syndrome virus (PRRSV) and positive submissions based on real-time RT-PCR testing for suckling pigs over the past 10 years. Figures were built using the number of submissions and positive submissions, where counts were obtained from swine samples submitted to the Animal Health Laboratory in Ontario from January 2014 to July 2023 for all case types. [file Image_3.tiff]

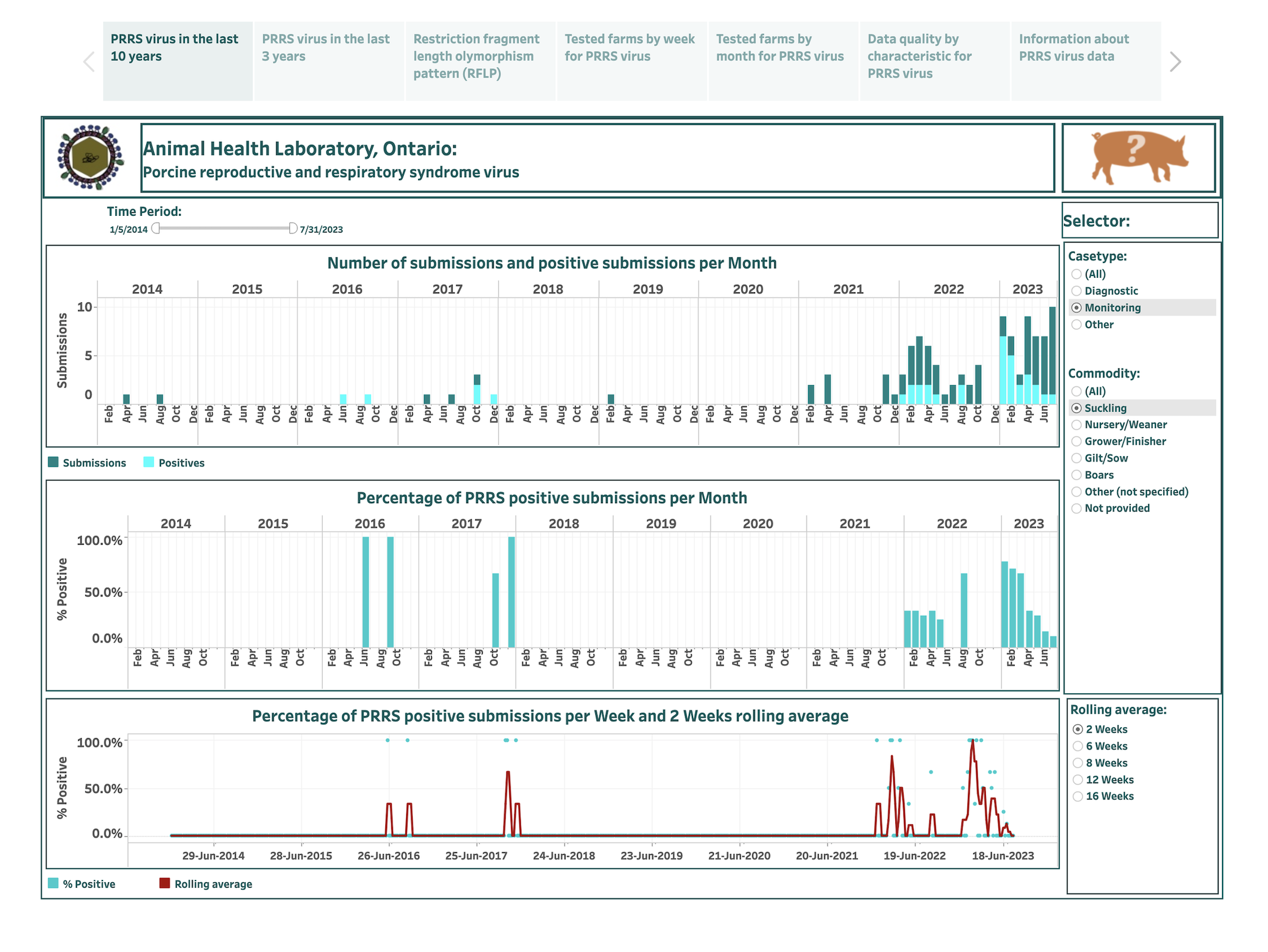

Supplement: Supplementary Figure 4 — Dashboard for monthly number of submissions for porcine reproductive and respiratory syndrome virus (PRRSV) and positive submissions based on real-time RT-PCR testing for suckling pigs over the past 10 years. Figures were built using the number of submissions and positive submissions, where counts were obtained from swine samples submitted to the Animal Health Laboratory in Ontario from January 2014 to July 2023. Charts display aggregated test results obtained from submissions tested for monitoring purpose. [file Image_4.tiff]

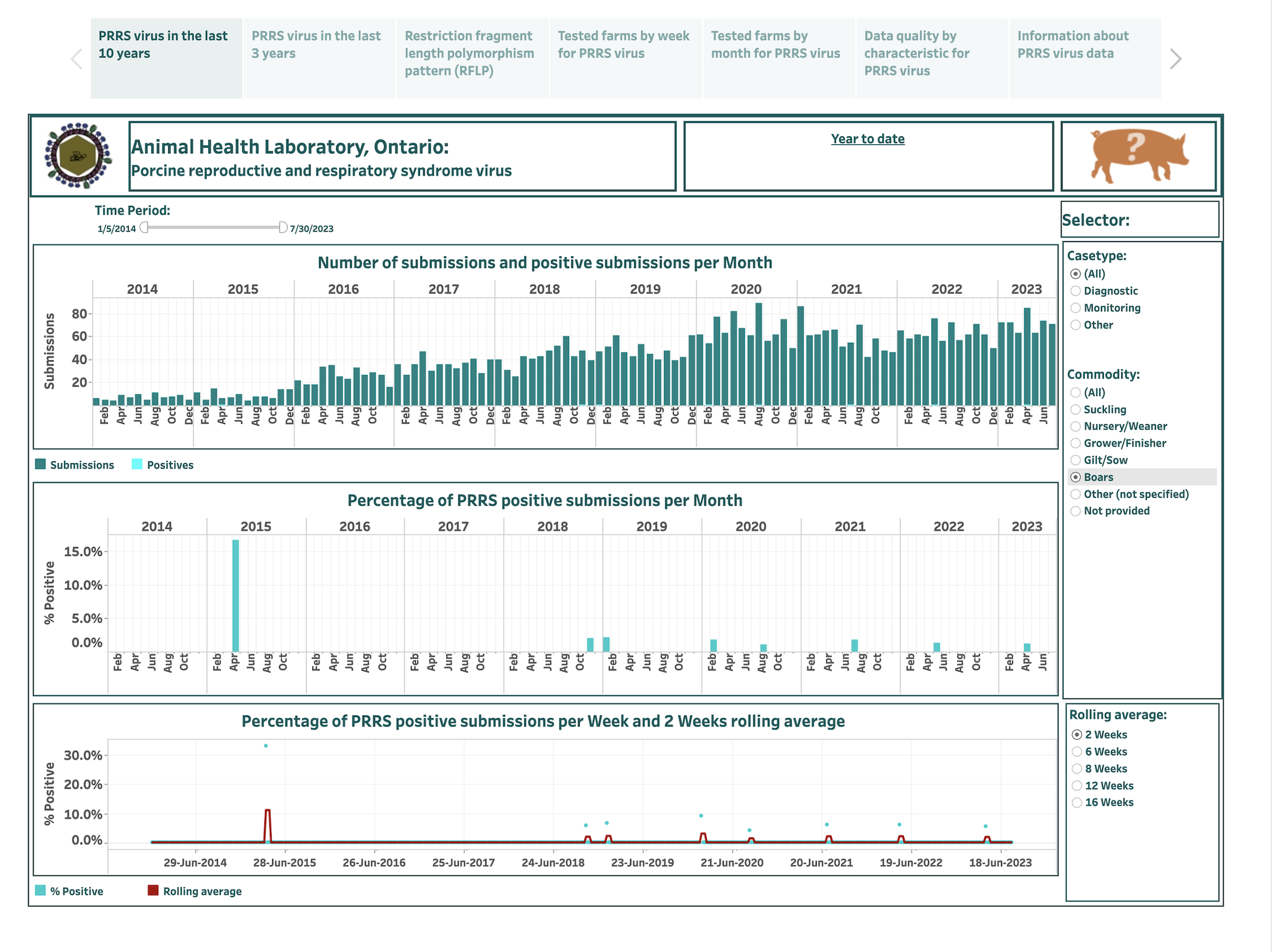

Supplement: Supplementary Figure 5 — Dashboard for monthly number of submissions for porcine reproductive and respiratory syndrome virus (PRRSV) and positive submissions based on real-time RT-PCR testing for boars over the past 10 years. Figures were built using the number of submissions and positive submissions, where counts were obtained from swine samples submitted to the Animal Health Laboratory in Ontario from January 2014 to July 2023 for all case types. [file Image_5.tiff]
